# Tranexamic Acid in Non-Traumatic Intracerebral Haemorrhage (TANICH II): Introducing the Potential Role of 3 g Tranexamic Acid in Haematoma Reduction

**DOI:** 10.21315/mjms2023.30.3.8

**Published:** 2023-06-27

**Authors:** Ananda Arumugam, Shze Ee Tan, Sze Ling Tan, Jun Ee Tan, Fatimah @ Hartina Hussin, Mohd Sofan Zenian, Zamzuri Idris, Jafri Malin Abdullah

**Affiliations:** 1 Department of Neurosurgery, Hospital Queen Elizabeth II, Sabah, Malaysia; 2 Department of Neurosurgery, Hospital Queen Elizabeth I, Sabah, Malaysia; 3 Department of Radiology, Hospital Queen Elizabeth I, Sabah, Malaysia; 4 Department of Pharmacy, Hospital Queen Elizabeth II, Sabah, Malaysia; 5 Department of Neurosciences, Brain and Behaviour Cluster, Hospital Universiti Sains Malaysia, Universiti Sains Malaysia, Kelantan, Malaysia

**Keywords:** tranexamic acid, optimal dose, haematoma reduction, intracerebral haemorrhage

## Abstract

**Background:**

Intracerebral haemorrhage (ICH) can be devastating, particularly if haematoma expansion occurs. The efficacy of tranexamic acid (TXA), an anti-fibrinolytic agent, in reducing haematoma expansion is now being studied worldwide. However, the optimal dosage of TXA has yet to be determined. This study was designed to further establish the potential of different doses of TXA.

**Methods:**

A double-blinded, randomised, placebo-controlled study was carried out among adults with non-traumatic ICH. Eligible study subjects were randomly assigned to receive placebo, 2-g TXA treatment or 3-g TXA treatment. Haematoma volumes before and after intervention were measured using the planimetric method.

**Results:**

A total of 60 subjects with 20 subjects in each treatment group were recruited for this study. Among the 60 subjects, the majority were male (*n* = 36, 60%), had known cases of hypertension (*n* = 43, 71.7%) and presented with full Glasgow coma scale (GCS) (*n* = 41, 68.3%). The results showed that there was no statistically significant difference (*P* = 0.315) in the mean changes of haematoma volume when compared with three study groups using ANCOVA, although the 3-g TXA group was the only group that showed haematoma volume reduction (mean reduction of 0.2 cm^3^) instead of expansion as in placebo (mean expansion 1.8 cm^3^) and 2-g TXA (mean expansion 0.3 cm^3^) groups. Good recovery was observed in all study groups, with only three subjects being moderately disabled. No adverse effects were reported in any of the study groups.

**Conclusion:**

To the best of our knowledge, this is the first clinical study using 3 g of TXA in the management of non-traumatic ICH. From our study, 3 g of TXA may potentially be helpful in reducing haematoma volume. Nonetheless, a larger-scale randomised controlled trial should be carried out to further establish the role of 3 g of TXA in non-traumatic ICH.

## Introduction

Intracerebral haemorrhage (ICH) results in high mortality and morbidity in the population (1). Haematoma expansion after acute ICH is common and is associated with early neurological deterioration (END) and poor clinical outcome (2). Clinical and biologic markers of the inflammatory reaction on admission are predictors of subsequent END, whereas early intracerebral haematoma growth, intraventricular bleeding and high systolic blood pressure (SBP) within 48 h are factors associated with END in patients with ICH (2). Several studies have investigated the potential therapies for ICH, many of which were designed to identify and treat ICH progression. Despite this, there is currently no approved, effective therapy (3, 4).

Early haematoma expansion in patients with ICH is a promising treatment target. To date, the time course of early haematoma expansion has remained poorly described. Ovesen et al. (5) studied the time course of early post-admission haematoma expansion in spontaneous ICH. Among 25 patients with ICH, 10 (40%) patients had early haematoma expansion (5). Early haematoma expansion was reliably reflected by transcranial B-mode ultrasound and mainly occurred within the first 7 h–8 h after symptom onset (5). The approaches to ICH have broadly focused on arresting haematoma expansion. Early intervention should include control of hypertension with intravenous beta-blockers or calcium channel blockers, especially when patients presenting with SBP > 220 mmHg, correction of any coagulopathy if present and assessment of the need for surgical intervention.

To date, there has been no evidence-based effective treatment for acute ICH (6–16). The *Tranexamic Acid in IntraCerebral Haemorrhage* (TICH) trial published by Sprigg et al. (17) recruited 24 patients within 24 h of ICH to receive either 1 g of tranexamic acid (TXA) or placebo over 10 min, followed by 1 g of TXA over 8 h; non-statistically significant haematoma expansion was noted in the TXA treatment group. This was followed by the *Tranexamic Acid for Intracerebral Haemorrhage-2* (TICH-2) trial (18), in which 2,325 participants were recruited, with 1,161 participants receiving TXA and 1,164 participants receiving placebo. Functional status at 90 days after ICH did not differ significantly between the study arms, despite a reduction in early death and serious adverse events in the TXA group. Sprigg et al. (18) suggested that larger randomised trials are needed to confirm or refute a clinically significant treatment effect.

Here locally, Arumugam et al. (19) conducted a study to assess the effect of TXA on haematoma growth in non-traumatic ICH compared to placebo and they concluded a significant haematoma growth in the control group, whereas in the TXA treatment group, there was no significant haematoma size expansion (19).

We aimed to further determine the advantages as well as the optimal dosage for the use of TXA in the management of non-traumatic ICH, along with strict SBP optimisation.

## Methods

This study was designed as a doubleblinded, randomised, placebo-controlled trial conducted in the emergency department of Hospital Queen Elizabeth I and II, Sabah, from 2016 to 2019. Simple random sampling was employed, whereas randomisation was done via 2 × 3 permuted blocks, with the randomisation list generated using www.randomization.com.

Inclusion criteria for this study were patients aged ≥18 years old (either gender) diagnosed with hypertensive ICH, which happened within 8 h of onset. The lesion should be around the supratentorial area and deemed inappropriate to be surgically removed by an on-call neurosurgeon. The exclusion criteria were as follows: ICH other than hypertensive causes; patient taking anticoagulants or antiplatelet; known case of blood disorders (e.g. haemophilia and idiopathic thrombocytopenic purpura); hepatic or renal impairment; infection; previous venous thrombosis or embolic disease; recent ischaemic event (within 12 months), such as ischaemic stroke, myocardial infarction or peripheral artery disease; and pregnant or breast-feeding women (pregnancy was excluded in women of child-bearing age using a urine pregnancy test).

During the study period, patients presented to the emergency department with the diagnosis of ICH were screened for eligibility for enrolment in this study. Routine investigations such as full blood count (FBC), renal profile (RP), liver function test (LFT), coagulation profile and computed tomography (CT) of the brain were done prior to the screening. Strict BP control (SBP < 140 mmHg–160 mmHg) was also initiated, as routine management, using labetalol infusion or other options of anti-hypertensives, whenever indicated.

Should the patient meet the inclusion/exclusion criteria, the patient and family (legalised authorised representative [LAR]) were given sufficient time to read through the patient’s information sheet for study recruitment by the investigator. A consent form was signed and dated once the patient and LAR agreed to participate in the trial. Patients were randomised according to the randomisation list into placebo (10 mL of normal saline [NS] as a slow bolus over 10 min followed by 100 mL of NS infusion over 8 h), 2-g TXA (1 g of TXA as a slow bolus over 10 min followed by 1 g of TXA infusion over 8 h) or 3-g TXA (1 g of TXA as a slow bolus over 10 min followed by 2 g of TXA infusion over 8 h) groups. TXA used in this trial was manufactured by Bioindustria L.I.M. S.p.A., Italy. In this study, the patients and the outcome assessor were unaware of the treatment, whereas the investigator was aware of the study allocation.

Thereafter, the recruited subjects were transferred to the neurosurgery ward for the continuation of care. All subjects were monitored for adverse events and hypersensitivity reactions, and they subsequently underwent a repeat CT brain after 24 h of observation. Subjects were discharged after recovery and reviewed again in 30 days to assess their general well-being, as well as the Glasgow outcome score (GOS) and modified Rankin Scale (mRS). A good outcome is defined as having GOS 4–5 or mRS 0–2 (20).

## Results

The main study outcome was to assess the efficacy of TXA in different dosages (3 g and 2 g) as compared to placebo concerning the prevention of haematoma expansion in hypertensive ICH. For haematoma outcome assessment, CT films taken for each recruited patient on admission and upon study completion were sent to the assigned radiologist for the measurement of haematoma volume via planimetric measurement technique. To avoid outcome assessment bias, the radiologist was not informed about the treatment allocation. Other study outcomes include the assessment of the difference of SBP and white blood cell (WBC) count at presentation versus 24 h, safety of TXA, and patient’s GOS and mRS at day 30 post-discharge.

### Sample Calculation

For the objective of comparing changes in haematoma size between the control and two treatment groups, a sample size of 51 for each group was required in order to detect the mean difference of 3.00 with a power of 0.80 (80%) and an alpha of 0.05. The mean difference of 3.00 was considered the smallest important difference to be detected. The SD of the variable of interest was estimated as 5.37 based on the pilot study done previously (19). This calculation was done using ScalexMean version 1.0.2 (20). Taking into account the 20% dropping rate, a total of 173 patients would have been recruited for this study. However, only 60 patients (20 patients in each group) were recruited at the end of the study due to slow recruitments over 4 years.

### Statistical Analysis

Descriptive statistics of proportion and mean were used to describe the patients’ demographic data. To investigate the effects of three study groups (placebo, 2 g TXA, and 3 g TXA) on haematoma size as well as to control the pre-treatment haematoma size (covariate), analysis of covariance (ANCOVA) was used. The changes in haematoma size, mean SBP, and mean WBC count on admission and after 24 h for each group were analysed using a paired *t*-test. A *P*-value of less than 0.05 is considered statistically significant in all inferential statistics. GOS and mRS at day 30 post-discharge, as well as the number of side effects experienced by the patient, were presented in proportion.

### Characteristics of Subjects

Due to slow recruitment, only 60 subjects were recruited in this study. Table 1 summarises the demographics of the subjects. Tables 2 and 3 summarise the clinical characteristics of all subjects in three study groups (*n* = 20 each). There were no statistically significant differences among all the demographics and clinical characteristics between the three study groups. Among the 60 subjects, the majority were male (*n* = 36, 60%), had known cases of hypertension (*n* = 43, 71.7%) and presented with full GCS (*n* = 41, 68.3%).

### Haematoma Volume

The mean haematoma volume on admission and at 24 h after admission among three study groups is shown in Table 4. Paired *t*-tests showed that there were no statistically significant changes in haematoma volume between pre- and post-treatments in all three study groups. In order to test the effects of three study groups on haematoma volume while controlling the pretreatment haematoma volume, ANCOVA was employed. The result has shown the *P*-value to be 0.315, which means that there is no statistically significant difference in the mean changes of haematoma volume among three study groups, despite the 3-g TXA group being the only group that showed numerically haematoma volume reduction instead of expansion as in placebo and 2-g TXA groups.

### SBP and Total White Blood Cell

The changes in SBP and total WBC count from admission and at 24 h after admission are shown in Table 5. It was noted that SBP at 24 h was statistically lower compared to SBP on admission in all three groups. As for total WBC count, there were no statistically significant changes between pre- and post-treatments in all three groups.

### Other Outcomes

Table 6 shows the other outcomes studied in this study. Good recovery was observed in all study groups, with only three subjects being moderately disabled. No adverse effects were reported in any of the study groups.

## Discussion

To the best of our knowledge, this is the first study looking into the potential of different dosages of TXA (2 g and 3 g) in preventing haematoma expansion in non-traumatic ICH. Overall, we noted that there was a numerical reduction of haematoma volume in the 3-g TXA group instead of expansion as seen in the 2-g TXA and placebo groups, although there were no statistically significant differences in the mean changes of haematoma volume between pre- and post-treatments across the three study groups (placebo, 2 g TXA and 3 g TXA).

To date, there has been no proven treatment to limit haematoma expansion in ICH (21). The current main non-surgical strategies have been postulated to reduce haematoma expansion: reduction of perfusion pressure (SBP) and haemostatic therapy (22). TXA is a promising haemostatic agent, given its proven efficacy in reducing bleeding rates as well as the need for blood transfusion in surgical and trauma patients (23–38). It belongs to a class of drugs known as anti-fibrinolytic. It is a synthetic derivative of the amino acid lysine, which binds five lysine binding sites on plasminogen. This inhibits plasmin formation and displaces the plasminogen from the fibrin surface. It may also directly inhibit plasmin and partially inhibit fibrinogenolysis at higher concentrations. In summary, it inhibits the dissolution of fibrin, thereby stabilising the clot and preventing further haemorrhage. Furthermore, TXA may have anti-inflammatory and neuroprotective effects by reducing perihematomal oedema and improving the healing process, manifesting as a reduction in haematoma cavity and surrounding gliosis (22).

Sorimachi et al. (40) conducted a retrospective observational study in 156 patients who received either 1 g of TXA over 6 h or 2 g of TXA over 10 min, and they reported less haematoma expansion in patients who received rapid TXA infusion (*P* = 0.001) (39). Ojacastro et al. (41) conducted another observational study in 30 patients who received one of three doses of 500 mg of TXA or usual care, and they reported less haematoma expansion in the TXA group (39). Sprigg et al. conducted the pilot RCT on TXA in ICH (TICH), which recruited 24 patients to receive either placebo or 1 g of TXA over 10 min, followed by 1 g of TXA infusion over 8 h, with non-significantly more haematoma expansion observed in the TXA arm (17). Our study finding, though not enough to draw any conclusion, is indeed hypothesis-generating, which warrants for further study on the potential of TXA in non-traumatic ICH, especially at a higher dosage. This finding was also consistent with the pilot TXA trial done locally by Arumugam et al. (19), in which significant haematoma expansion was observed in the placebo group.

In terms of SBP reduction, our study has shown that aggressive SBP reduction can be achieved by using various types of parenteral or oral anti-hypertensives. Mean SBP among three study groups was statistically lower (mean SBP < 140 mmHg) in patients at 24 h after admission than in those on admission. The intensive blood pressure reduction in acute cerebral haemorrhage trial (INTERACT) reported a reduction in haematoma expansion at 24 h in patients treated with intensive BP reduction (SBP < 140 mmHg), as compared to standard BP reduction (SBP < 180 mmHg) (10). This was further supported by the INTERACT-2 trial, which suggested that an SBP of 130–139 mmHg was likely to give maximum benefit in moderately sized ICH (11). The American Heart Association (AHA) guideline, similarly, recommended that for ICH patients presenting with SBP between 150 mmHg and 220 mmHg, and without contraindication to acute BP treatment, lowering of SBP to 140 mmHg is safe (12, 42). The antihypertensive treatment of acute cerebral haemorrhage (ATACH-2) trial, however, did not find a reduction in haematoma expansion with intensive SBP lowering (8, 43). Our study did not find significant evidence that SBP reduction leads to a reduction of haematoma volume, possibly related to inclusion criteria of smaller haematoma volume.

Apart from SBP reduction and TXA therapy, the relevance of leucocyte counts to ICH prognosis has also attracted mounting attention, but no consensus has been reached (5). Although chronic inflammation mediates ICH-induced secondary brain injury, acute inflammation reaction is beneficial for the early benign transformation of the haematoma. A procoagulant state induced by acute inflammation may limit haematoma enlargement. Chen et al. (44) conducted a retrospective analysis to find an association between eosinophilic leucocyte count and haematoma expansion in ICH. They revealed that the risk of haematoma expansion was inversely associated with neutrophil counts and directly associated with monocyte counts (44). It was also noted that eosinophil counts were robustly correlated with haematoma expansion irrespective of the definition of haematoma expansion. However, in our study, there was no change in mean WBC count between pre- and post-treatments in the three study groups.

In our study, all study subjects were followed up with GOS and mRS assessment at 30 days after ICH, as evidence reported a median 1-month mortality rate of 40%. A local pilot TXA study by Arumugam et al. (19) used GOS as the primary outcome measure. Thus, in this study, we kept GOS for comparison purposes and added mRS assessment as the additional functional outcome measure. In our trial, good recovery scores were recorded in all study subjects, with 95% of our study subjects managing to achieve fair independence in their daily living.

## Conclusion

To the best of our knowledge, this is the first clinical study using 3 g of TXA in the management of non-traumatic ICH. From our observation, 3 g of TXA appeared to be potentially helpful in reducing haematoma volume, with no adverse reaction reported, possibly due to the better efficacy of TXA in stabilising the fibrin network at a higher dosage. Therefore, we suggest that a 3-g TXA regime should be further studied to establish a safe and beneficial therapy in patients with non-traumatic ICH.

## Limitations

Due to time constraints as well as slow recruitment, we were unable to achieve the number of subjects needed as per the sample size calculation. In addition, this study was only a single-centre study, which may limit the generalisability of the results. Despite the limitation, given the potential benefits and lack of adverse effects noted in this study, future research targeting a larger study population or multicentre recruitment may provide stronger evidence in establishing the role of 3-g TXA treatment in non-traumatic ICH.

## Figures and Tables

**Table 1:** Demographics of subject

Variables		Placebo *n* = 20	2 g TXA *n* = 20	3 g TXA *n* = 20	*P*-value
Age, in years old (mean, standard deviation)		52.9 (10.05)	51.6 (11.73)	52.4 (9.56)	0.857$
Gender, *n* (%)	Male	12 (60.0)	11 (55.0)	13 (65.0)	0.812*
	Female	8 (40.0)	9 (45.0)	7 (35.0)	
Co-morbidity, *n* (%)	Hypertension	16 (80.0)	15 (75.0)	12 (60.0)	0.344*
	Dyslipidemia	2 (10.0)	2 (10.0)	1 (5.0)	> 0.950#
	Diabetes mellitus	4 (20.0)	1 (5.0)	1 (5.0)	0.344#

Note:

$ one-way ANOVA;

* chi-square test;

# Fisher’s exact test;

TXA = Tranexamic acid

**Table 2:** Clinical characteristics of subjects (*n* = 60), categorical variables

Variables, in categorical, *n* (%)		Placebo *n* = 20	2 g TXA *n* = 20	3 g TXA *n* = 20	*P*-value
GCS at presentation	9–12	3 (15.0)	7 (35.0)	5 (25.0)	0.434#
	13–14	2 (10.0)	0 (0.0)	2 (10.0)	
	15	15 (75.0)	13 (65.0)	13 (65.0)	
Location of haematoma	Putamen	16 (80.0)	15 (75.0)	16 (80.0)	0.416#
	Thalamus	1 (5.0)	4 (20.0)	3 (15.0)	
	Subcortex	2 (10.0)	0 (0.0)	0 (0.0)	
	Other	1 (5.0)	1 (5.0)	1 (5.0)	
Shape	Irregular	11 (55.0)	11 (55.0)	6 (30.0)	0.130#
	Regular	9 (45.0)	9 (45.0)	14 (70.0)	
Anti-hypertensive used during acute presentation	IVI labetalol	15 (75.0)	15 (75.0)	16 (80.0)	>0.950#
	IVI Hydralazine	2 (10.0)	2 (10.0)	2 (10.0)	>0.950#
	ACEi	8 (40.0)	6 (30.0)	11 (55.0)	0.833*
	CCB	10 (50.0)	13 (65.0)	16 (80.0)	0.488*

Notes:

# Fisher’s exact test;

* chi-square test;

TXA = Tranexamic acid; GCS: Glasgow coma score; IVI = intravenous infusion; ACEi = angiotensin-converting enzyme inhibitor; CCB = calcium channel blocker

**Table 3:** Clinical characteristics of subjects (*n* = 60), numerical variables

Variables, in numerical, mean (SD)	Placebo *n* = 20	2 g TXA *n* = 20	3 g TXA *n* = 20	*P*-value
Duration of 1st CT brain from symptom onset (min)	236.5 (160.27)	161.5 (117.95)	241.6 (131.71)	0.129$
Haemoglobin (g/L)	14.5 (1.70)	14.6 (1.76)	13.5 (1.80)	0.080$
Platelet count (10^3^/uL)	303.3 (87.12)	269 (59.70)	295 (91.87)	0.373$
Prothrombin time (s)	12.4 (1.66)	11.8 (1.33)	12.0 (1.00)	0.300$
Activated partial thromboplastin time (s)	33.6 (6.38)	30.7 (6.18)	34.3 (6.44)	0.164$
International normalised ratio	1.0 (0.09)	1.0 (0.06)	1.0 (0.08)	0.923$

Note:

$ one-way ANOVA;

TXA = Tranexamic acid

**Table 4:** Mean (standard deviation) haematoma volume (cm^3^) on admission and at 24 h for three study groups

Treatment group	On admission	24 h	Mean difference (95% CI)	*t* (df)	*P*-value
Placebo, *n* = 20	10.7 (7.16)	12.4 (12.02)	1.8 (−1.06, 4.62)	1.31 (19)	0.206
2 g TXA, *n* = 20	11.1 (7.28)	11.5 (8.03)	0.3 (−1.27, 1.93)	0.43 (19)	0.672
3 g TXA, *n* = 20	10.3 (6.03)	10.1 (5.96)	−0.2 (−1.39, 1.02)	−0.32 (19)	0.751

Note: TXA = Tranexamic acid

**Table 5:** Systolic blood pressure and total white blood cell on admission and 24 h

Variable	Treatment group	On admission	24 h	Mean difference (95% CI)	*t* (df)	*P*-value
SBP (mmHg)	Placebo *n* = 20	187.9 (52.40)	125.1 (14.44)	−62.9 (−86.18, −39.52)	5.64 (19)	< 0.001
	2g TXA *n* = 20	196.5 (20.65)	127.7 (20.53)	−68.8 (−80.04, −57.46)	12.75 (19)	< 0.001
	3g TXA *n* = 20	194.7 (29.76)	124.9 (17.22)	−71.8 (−87.40, −56.20)	0.32 (19)	< 0.001
WBC Count (10^3^/uL)	Placebo, *n* = 20	10.2 (3.17)	10.2 (3.04)	−0.1 (−1.59, 1.41)	0.13 (19)	0.896
	2g TXA, *n* = 20	11.2 (2.20)	11.1 (3.44)	−0.02 (−1.39, 1.35)	0.03 (19)	> 0.950
	3g TXA, *n* = 20	9.9 (2.76)	10.1 (2.29)	0.3 (−1.35, 1.84)	−0.34 (19)	0.740

Notes: SBP = systolic blood pressure; WBC = white blood cell; TXA = Tranexamic acid

**Table 6:** Other outcomes studied

Outcome, *n* (%)		Placebo, *n* = 20	2g TXA, *n* = 20	3g TXA, *n* = 20
GOS score at day 30	1–3	2 (10.0)	1 (5.0)	0 (0.0)
	4–5	18 (90.0)	18 (90.0)	20 (100.0)
mRS score at day 30	0–3	18 (90.0)	18 (90.0)	20 (100.0)
	4–6	2 (10.0)	1 (5.0)	0 (0.0)
Adverse effect		0 (0.0)	0 (0.0)	0 (0.0)

Notes: TXA = Tranexamic acid; GOS = Glasgow outcome score; mRS = Modified Rankin scale

## References

[1] Aziz ZA, Lee YY, Ngah BA, Sidek NN, Looi I, Hanip MR (2015). Acute stroke registry Malaysia, 2010–2014: results from the National Neurology Registry. J Stroke Cerebrovasc Dis.

[2] Leira R, Dávalos A, Silva Y, Gil-Peralta A, Tejada J, Garcia M (2004). Early neurologic deterioration in intracerebral haemorrhage: predictors and associated factors. Neurology.

[3] Garg R, Biller J (2019). Recent advances in spontaneous intracerebral hemorrhage. F1000Research.

[4] Specogna AV, Turin TC, Patten SB, Hill MD (2014). Factors associated with early deterioration after spontaneous intracerebral hemorrhage: a systematic review and meta-analysis. PLoS One.

[5] Ovesen C, Christensen AF, Krieger DW, Rosenbaum S, Havsteen I, Christensen H (2014). Time course of early post admission hematoma expansion in spontaneous intracerebral haemorrhage. Stroke.

[6] Manios E, Gasecki D, Coca A, Cunha P, Hering D, Lovic D (2017). Blood pressure targets in acute intracerebral hemorrhage. Eur Soc Hypertens.

[7] Mule G, Sorce A, Giambrone M, Fierro B, Cottone S, Cerasola G (2019). The unsolved conundrum of optimal blood pressure target during acute hemorrhage stroke: a compressive analysis. High Blood Press Cardiovasc Prev.

[8] Pan C, Hu Y, Liu N, Zhang P, Zhang YP, Aimaiti M (2015). Aggressive blood pressure lowing therapy in patients with acute intracerebral haemorrhage is safe: a systematic review and meta-analysis. Chin Med J.

[9] Qureshi AI, Tariq N, Divani AA, Novitzke J, Hussein HH, Palesch YY (2010). Antihypertensive treatment of acute cerebral hemorrhage (ATACH). Crit Care Med.

[10] Anderson CS, Huang Y, Wang JG, Arima H, Neal B, Peng B (2008). Intensive blood pressure reduction in acute cerebral haemorrhage trial (INTERACT). A randomized pilot trial. Lancet Neurol.

[11] Arima H, Heeley E, Delcourt C, Hirakawa Y, Wang X, Woodward M (2015). Optimal achieved blood pressure in acute intracerebral haemorrhage: INTERACT2. Neurology.

[12] Hemphill JC (2019). Hematoma expansion in ICH: targeting epidemiology or biology?. Neurocrit Care.

[13] Ducloy-Bouthors AS, Duhamel A, Kipnis E, Tournoys A, Prado-Dupont A, Elkalioubie A (2016). Postpartum haemorrhage related early increase in D-dimers is inhibited by tranexamic acid: haemostasis parameters of a randomized controlled open labelled trial. Br J Anaesth.

[14] Nishihara S, Hamada M (2015). Does tranexamic acid alter the risk of thromboembolism after total hip arthroplasty in the absence of routine chemical thromboprophylaxis?. Bone Joint J.

[15] Rabinstein AA (2018). Optimal blood pressure after intracerebral hemorrhage. Stroke.

[16] Cusack TJ, Carhuapoma JR, Ziai WC (2018). Update on the treatment of spontaneous intraparenchymal hemorrhage: medical and interventional management. Curr Treat Options Neurol.

[17] Sprigg N, Renton CJ, Dineen RA, Kwong Y, Bath PM (2014). Tranexamic acid for spontaneous intracerebral haemorrhage: a randomized controlled pilot trial. J Stroke Cerebrovasc Dis.

[18] Sprigg N, Flaherty K, Appleton JP, Salman RAS, Bereczki D, Beridze M (2018). Tranexamic acid for hyperacute primary intracerebral haemorrhage (TICH-2): an international randomised, placebo-controlled, phase 3 superiority trial. Lancet.

[19] Arumugam A, A Rahman NA, Theophilus SC, Shariffudin A, Abdullah JM (2015). Tranexamic acid as antifibrinolytic agent in non-traumatic intracerebral haemorrhages. Malays J Med Sci.

[20] Lin Naing ScalexMean version 1.0.2. Sample size calculator for comparing two means (Inequality).

[21] Tong JT, Eyngorn I, Mlynash M, Albers GW, Hirsch KG (2017). Functional neurologic outcomes change over the first six months after cardiac arrest. Crit Care Med.

[22] Lees KR, Bath PMW, Schellinger PD, Kerr DM, Fulton R, Hacke W (2012). Contemporary outcome measures in acute stroke research. choice of primary outcome measures. Stroke.

[23] Cook R, Lyon-Maris J, Martin R, NIHR Dissemination Centre (2020). Tranexamic acid is safe to use following mild-to-moderate traumatic brain injury. BMJ.

[24] CRASH-2 Collaborators (Intracranial Bleeding Study) (2011). Effect of tranexamic acid in traumatic brain injury: a nested randomised, placebo controlled trial (CRASH-2 intracranial bleeding study). BMJ.

[25] Roberts I, Shakur H, Coats T, Hunt B, Balogun E, CRASH-2 Collaborators (2011). The importance of early treatment with tranexamic acid in bleeding trauma patients: an exploratory analysis of the CRASH-2 randomised controlled trial. Lancet.

[26] CRASH-3 Trials Collaborators (2019). Effects of tranexamic acid on death, disability, vascular occlusive events and other morbidities in patients with acute traumatic brain injury (CRASH-3): a randomised, placebo-controlled trial. Lancet.

[27] Estcourt LJ, Desborough M, Brunskill SJ, Doree C, Hopewell S, Murphy MF (2016). Antifibrinolytics (lysine analogues) for the prevention of bleeding in people with haematological disorders. Cochrane Database Syst Rev.

[28] Garg J, Pinnamaneni S, Aronow WS, Ahmad H (2014). ST elevation myocardial infarction after tranexamic acid: first reported case in the United States. Am J Ther.

[29] Menkis AH, Martin J, Cheng DC, Fitzgerald DC, Freedman JJ, Gao CQ (2012). Drug, devices, technologies, and techniques for blood management in minimally invasive and conventional cardiothoracic surgery: a consensus statement from the International Society for Minimally Invasive Cardiothoracic Surgery (ISMICS) 2011. Innovations.

[30] Murphy GR, Glass GE, Jain A (2016). The efficacy and safety of tranexamic acid in cranio-maxillofacial and plastic surgery. J Craniofac Surg.

[31] Roberts I, Belli A, Brenner A, Chaudhri R, Fawole B, Harris T (2018). Tranexamic acid for significant traumatic brain injury (the CRASH-3 trial): statistical analysis plan for an international, randomised, double-blind, placebo-controlled trial. Wellcome Open Res.

[32] Sanz-Reig J, Parra Ruiz B, Ferrández Martínez J, Martínez López JF (2016). Single intravenous tranexamic acid dose to reduce blood loss in primary total knee replacement. Rev Esp Cir Ortop Traumatol.

[33] Sentilhes L, Daniel V, Darsonval A, Deruelle P, Vardon D, Perrotin F (2015). Study protocol. TRAAP—tranexamic acid for preventing postpartum hemorrhage after vaginal delivery: a multicenter randomized, double-blind, placebo-controlled trial. BMC Pregnancy Childbirth.

[34] Sigaut S, Tremey B, Ouattara A, Couturier R, Taberlet C, Grassin-Delyle S (2014). Comparison of two doses of tranexamic acid in adults undergoing cardiac surgery with cardiopulmonary bypass. Anaesthesiology.

[35] Vel R, Udupi BP, Satya Prakash MV, Adinarayanan S, Mishra S, Babu L (2015). Effect of low dose tranexamic acid on intra-operative blood loss in neurosurgical patients. Saudi J Anaesth.

[36] Volquind D, Zardo RA, Winkler BC, Londero BB, Zanelatto N, Leichtweis GP (2016). Use of tranexamic acid in primary total knee replacement: effects on perioperative blood loss. Braz J Anesthesiol.

[37] Wei Z, Liu M (2015). The effectiveness and safety of tranexamic acid in total hip or knee arthroplasty: a meta-analysis of 2720 cases. Transfus Med.

[38] Zehtabchi S, Abdel Baki SG, Falzon L, Nishijima DK (2014). Tranexamic acid for traumatic brain injury: a systematic review and meta-analysis. Am J Emerg Med.

[39] Law ZK, Meretoja A, Engelter ST, Christensen H, Muresan EM, Glad SB (2017). Treatment of intracerebral haemorrhage with tranexamic acid—a review of current evidence and ongoing trials. Eur Stroke J.

[40] Sorimachi T, Fujii Y, Morita K (2005). Rapid administration of antifibrinolytics and strict blood pressure control for intracerebral hemorrhage. Neurosurgery.

[41] Ojacastro MF, Tabuena MP, Dulos ID (2008). Efficacy of tranexamic acid in reducing hematoma volume in patients with hypertensive intracerebral hemorrhage. Int J Stroke.

[42] Hemphill JC, Greenberg SM, Anderson CS, Becker K, Bendok BR, Cushman M (2015). Guidelines for the management of spontaneous intracerebral haemorrhage: a guideline for healthcare professionals from the American Heart Association/American Stroke Association. Stroke.

[43] Qureshi AI, Palesch YY, Barsan WG, Hanley DF, Hsu CY, Martin RL (2016). Intensive blood pressure lowering in patients with acute cerebral hemorrhage. N Engl J Med.

[44] Chen Q, Liu J, Xu H, He W, Li Y, Jiao L (2019). Association between eosinophilic leukocyte count and hemataoma expansion in acute spontaneous intracerebral hemorrhage. Front Neurol.

